# Clinical Outcomes in Patients with Cystic Fibrosis-Related Chronic Rhinosinusitis Treated with Functional Endoscopic Sinus Surgery or Triple Highly Effective Modulator Therapy: A Monocentric Retrospective Experience

**DOI:** 10.3390/jcm14186498

**Published:** 2025-09-15

**Authors:** Pietro Orlando, Alberto Minzoni, Luca Mazzetti, Angelo Ricchiuti, Silvia Bresci, Giandomenico Maggiore

**Affiliations:** 1 Department of Otolaryngology, Careggi University Hospital, 50134 Florence, Italy; 2 Infectious and Tropical Diseases Unit, Careggi University Hospital, 50134 Florence, Italy

**Keywords:** cystic fibrosis, sense of smell, chronic rhinosinusitis, functional endoscopic sinus surgery, triple highly effective modulator therapy

## Abstract

**Background**: Cystic Fibrosis (CF) is an autosomal recessive inherited disease caused by mutations of the CF–transmembrane conductance regulator (CFTR), leading to impaired chloride ion trafficking, thickened secretions, and chronic rhinosinusitis (CF-CRS). CF-CRS was historically managed with intranasal corticosteroids (INCS) and endoscopic sinus surgery (ESS). Nowadays, the triple highly effective modulator therapy elexacaftor–tezacaftor–ivacaftor (ETI) is showing promising results in improving CF-CRS. **Methods**: This is a monocentric, retrospective study comparing Sinonasal Outcome Test-22 (SNOT-22), Nasal Polyps Score (NPS), modified Lund–Kennedy score (mLKS), sniffin’ sticks identification test (SSIT), and Lund–Mackay score (LMS) in patients affected by CF-CRS and treated with ESS or ETI. ETI patients were further subdivided based on previous surgery. **Results**: A total of 25 patients were surgically treated, and 54 were treated with ETI (specifically, 17 surgically naïve and 37 post-FESS patients). Patients undergoing ESS and those receiving ETI experienced statistically significant improvements in SNOT-22, SSIT, and LMS with no differences between groups and regardless of genetic or demographic characteristics. Conversely, ESS patients experienced significantly higher mean changes in NPS and mLKS. **Conclusions**: ETI and FESS were safe and effective in reducing the symptomatologic burden of CF-CRS. Even in the ETI epoch, surgery may play a crucial role in managing CF-CRS, particularly in patients not eligible for ETI or experiencing severe disease not adequately controlled with medical therapy alone.

## 1. Introduction

Cystic Fibrosis (CF) is an autosomal recessive inherited disease caused by mutations in both alleles of the CF gene. The CF gene is located on chromosome 7 and encodes a transmembrane chloride ion channel, known as the CF–transmembrane conductance regulator (CFTR) [1]. The deletion of the phenylalanine at codon 508 (ΔF508) is the most common defect, accounting for more than 70% of cases in the Caucasian population [2,3]. Mutant CFTR channels severely impair ion trafficking in epithelial secretory cells, disrupting fluid and electrolyte balance and leading to the thickening of secretions [4]. CF affects the lungs, pancreas, liver, sweat glands, vas deferens, and sinonasal mucosa [5]. CF-related Chronic Rhinosinusitis (CF-CRS) has progressively been recognized as a distinct diagnostic entity and classified as a secondary CRS as per EPOS2020 [6]. CF-CRS may significantly impact the quality of life and influence pulmonary health in affected patients, and it is estimated to affect up to 90% of CF patients, although only 10–55.8% complain of symptoms such as nasal obstruction, facial pressure, post-nasal drip, and hyposmia [7,8,9,10,11]. Managing CF-CRS may be challenging, involving topical antibiotics, nasal irrigations, intranasal corticosteroids (INCS), endoscopic sinus surgery (ESS), and CFTR modulator treatment [7,12,13]. The recent approval of the triple combination of CFTR corrector and potentiator, consisting of elexacaftor, tezacaftor, and ivacaftor (ETI), marked a milestone in the treatment of patients with the ΔF508 mutation, demonstrating positive effects on pulmonary and sinonasal function [14,15]. Before the widespread availability of the ETI, ESS was strongly recommended for patients with poorly controlled or refractory CF-CRS symptoms despite local medical treatment and to eradicate Gram-negative bacteria such as *Pseudomonas aeruginosa* [16]. As of today, there is a lack of evidence regarding the role of ESS and ETI in the management of CF-CRS, as well as their potential combination and optimal timing. Our study aimed to analyze sinonasal outcomes in patients affected by CF-CRS and treated with ESS and ETI. In addition, patients receiving ETI were further subdivided into two groups according to their history of previous sinonasal surgery.

## 2. Materials and Methods

This is a retrospective monocentric study. Data were collected by reviewing electronic medical records of patients > 18 years old and followed up at our tertiary university center between 2019 and 2024. We enrolled patients with the ΔF508/ΔF508 or ΔF508/minimal function variants that were diagnosed with CF-CRS, including CRS with nasal polyps (CRSwNP) and CRS sine nasal polyps (CRsNP). CF-CRS was diagnosed by combining the clinical history and the sinonasal videoendoscopic exam of the sinonasal cavities performed by an ENT specialist. We collected demographics (sex, age, smoking habit, airborne allergies, history of lung transplantation, history of previous ESS), genetics (heterozygous or homozygous for the ΔF508 mutation), and clinical data. CF-CRS burden was evaluated before and 12 months after treatment initiation. We noticed possible post-surgical complications and how they were treated. We investigated the sinonasal-related quality of life using the sinonasal outcome test-22 questionnaire (SNOT-22). The local burden of the disease was examined by a videoendoscopic exam of the sinonasal fossae and classified according to the nasal polyps score (NPS) and the modified Lund–Kennedy score (mLKS). NPS only included nasal polyps size discrimination in regard to the middle turbinate, while mLKS considers nasal polyps size, the severity of nasal edema, and the quality and quantity of nasal secretion [17,18]. The sense of smell was studied by a 16-pen sniffin’ sticks identification test (SSIT) [ODOFIN, Burghart Messtechnik GmbH, Holm, Germany ©]: a score ≥ 12/16 was considered normosmia, while a score ≤ 7/16 was considered anosmia; intermediate scores (8–11) were considered hyposmia [19]. Opacification of sinuses and nasal cavities at the CT scan was staged as per the Lund–Mackay score (LMS) [20]. All patients under ETI obtained a pulmonary evaluation, including functional test (FEV1, FVC, and Tiffeneau index) and a bronchoalveolar lavage before starting ETI and after 12 months.

Patients were subdivided into 3 groups, based on their current treatment:

- Group 1, including patients who underwent ESS, followed by nasal washings and INCS (1 puff per nostril twice daily);
- Group 2, including patients receiving ETI, nasal washings, and INCS (1 puff per nostril twice daily). They were further subdivided into group 2A, including surgically naïve patients, and group 2B, including patients who started ETI after at least 1 previous ESS.

A comparative analysis between pre-treatment and post-treatment scores was performed. Finally, therapeutic strategies, ESS and ETI, were compared, as well as outcomes between surgically naïve and post-ESS patients receiving ETI. Descriptive and mean values were calculated using Microsoft Excel 2019. The Shapiro–Wilk test was used to check for the normality of data. The unpaired two-tailed Student’s *t*-test and the Mann–Whitney test (for normally and non-normally distributed variables, respectively) were used to compare the mean scores for each item between subgroups. The paired *t*-test and the Wilcoxon signed-rank test were used to compare the scores between the first and the follow-up visit. The statistical analyses were performed with SPSS software (IBM Corp. Released 2020. IBM SPSS Statistics for Macintosh, Version 27.0. Armonk, NY, USA: IBM Corp). All statistical tests were two-sided, and a *p*-value of less than 0.05 was considered statistically significant.

## 3. Results

Overall, 79 patients were enrolled in the present study. Specifically, 25 patients were assigned to group 1 and 54 to group 2 (17 in 2A and 37 in 2B). Demographic, genetic, and clinical data for each group are summarized in Table 1. Of the patients, 49 (62.0%) had CF-CRSwNP and 31 (38.0%) had CF-CRSsNP. The entire cohort consisted of 45 men (57.0%) and 34 women (43.0%), with a mean age of 36.3 years (19–62). The majority of patients carried a heterozygous ΔF508 mutation (75.9%). 10 patients (12.7%) had a history of lung transplantation, while 72 patients (91.1%) had exocrine pancreatic insufficiency, and 31 (39.2%) had diabetes requiring insulin therapy. A total of 53 patients had a history of ≥1 previous surgery (67.1%), with a mean number of surgeries = 1.3. The mean age at first and last ESS was 21.9 (6–44) and 34.7 (17–60) years, respectively.

At baseline, patients who underwent surgery had a more severe NPS, mLKS, and SSIT, while patients under ETI had a worse SNOT-22 and, in particular, patients in group 2B had a significantly lower SNOT-22 and LMS compared to surgically naïve ones. On the contrary, no differences were highlighted in terms of demographic characteristics between the three groups. Analyses of the SNOT-22, NPS, SSIT, and LMS revealed that patients undergoing ESS and those receiving ETI experienced statistically significant improvements in their perceived quality of life, sense of smell, and radiological opacification of sinonasal fossae. On the contrary, only patients who underwent surgery showed a significant reduction in NPS. Furthermore, within the ETI cohort, a decrease in NPS was noted exclusively among post-ESS patients, whereas surgically naïve individuals did not show comparable improvement. Mean sinonasal scores at baseline and after one year of treatment, along with the mean variations, are displayed in Table 2. Subgroup analysis revealed significant differences in endoscopic outcomes between surgically treated patients and those receiving ETI alone. Specifically, ETI patients exhibited significantly lower changes in NPS and mLKS scores after 12 months, indicating less improvement in endoscopic disease severity. In contrast, no significant differences were observed between the two groups in terms of SNOT-22 scores, olfactory function, or LMS. Furthermore, no statistically significant differences emerged when comparing groups 2A and 2B. However, post-ESS patients showed a greater, albeit not statistically significant, mean improvement in SNOT-22 scores compared to surgically naïve patients after 12 months of ETI therapy. Surprisingly, no anamnestic, genetic, or demographic characteristics were found to impact the outcomes of surgically or medically treated patients, including the diagnosis of CRSwNP or CRSsNP. Comparisons between the three study groups are displayed in Table 3. No further analyses were conducted regarding the interval between surgery and the initiation of ETI, as only one patient had undergone surgery within five years before starting ETI. Only 2 patients from group 2B required revision surgery throughout the study period due to a frontoethmoidal mucocele in both cases. No postoperative complications were documented during the follow-up period in the ESS cohort and in the two ETI patients who required rescue surgery.

## 4. Discussion

CF is a prevalent life-limiting disease that affects multiple organs, notably the respiratory and gastrointestinal tracts. However, a large portion of patients experience a more or less severe sinonasal symptomatology that may heavily blemish their quality of life [6,7,8]. Due to the pathophysiology of the disease, the management of CF-CRS may be difficult, requiring a multidisciplinary approach and a combination of local, systemic, and surgical options [7]. Patients complaining of CF-CRS have been historically treated with nasal rinses, INCS, topical antibiotics, and dornase alfa, followed by ESS in case of inadequate symptom control with medical therapy [7,21]. The first report about CF-CRS and surgery dates back to 1991 [22]. From then on, several studies highlighted the safety and efficacy of ESS in improving SNOT-22 and LMS, as well as pulmonary function [22,23,24,25,26,27]. The recent introduction of ETI represented a revolution in the management of patients affected by CF, as it showed promising results in improving sinonasal-related quality of life, endoscopic features of sinonasal fossae, radiological opacification of paranasal sinuses, and sense of smell [28,29,30,31,32,33,34,35,36,37,38,39]. Furthermore, a drastic reduction in rhinology healthcare utilization following the initiation of ETI has been reported, and even in our population, only two patients under modulator therapy required revision sinonasal surgery due to complications from previous surgery (4.1%) [37].

Our results are in line with those reported in the literature, as we demonstrated the effectiveness of both surgery and ETI in enhancing quality of life, NPS, mLKS, and LMS. Regarding olfactory function, the current literature presents ambiguous findings. The majority of studies about CF-CRS and sense of smell following ESS reported subjective improvement in the sense of smell [28,40,41,42]. Similarly, most papers documented minimal or no improvement in olfactory function following ETI therapy, which contrasts with our previous findings, showing an increase in the proportion of normosmic patients from 26.7% to 77.8% after one year of ETI treatment [36,43,44]. As expected, surgically treated patients exhibited greater mean NPS and mLKS variations than ETI patients after one year, while no differences were found in terms of SNOT-22 and SSIT. On the contrary, no differences were found when comparing post-ESS and surgically naïve patients under ETI. Sinonasal-related quality of life is a heterogeneous and multifaceted concept, encompassing rhinological, sleep, and psychological domains. Similarly, the sense of smell is a complex system requiring empty nasal cavities, healthy mucus lining the sinonasal mucosa, and functioning receptors and nerves. ESS may remove nasal polyps and enlarge natural sinus ostia. On the other hand, ETI acts systematically by improving mucus viscosity, cilia beating frequency, and pro-inflammatory cytokines concentration [38,39,40]. Therefore, both surgical and medical strategies may variably influence airflow, mucus drainage, nasal obstruction and congestion, bacterial superinfection, and odor molecules delivery to the olfactory cleft and their adhesion to the neuroepithelium. We may speculate that ESS may serve as a preparatory intervention that optimizes the sinonasal environment for subsequent ETI therapy by improving nasal patency and mucus drainage. However, we observed no statistically significant differences in clinical outcomes between patients who had undergone ESS and those who were surgically naïve at the time of ETI initiation. As such, our findings do not support the conclusion that prior surgery either enhances or compromises the efficacy of ETI. Finally, we were unable to determine the optimal timing for a possible sequential ESS–ETI treatment strategy, due to the lack of complete data regarding the interval between the most recent surgery and the initiation of ETI. It is worth noting that only one patient in our cohort initiated ETI within five years of undergoing ESS, limiting our ability to thoroughly assess the short-term impact of surgery on subsequent ETI outcomes. It is plausible that the clinical benefits of ESS may vary depending on the timing of subsequent ETI therapy. Unfortunately, data on the interval between the most recent ESS and ETI initiation are incomplete, and our medical records lack sufficient cases of patients who began ETI within a shorter-than-five-year timeframe following surgery. As a result, we were unable to investigate the potential relationship between the surgery-to-ETI interval and clinical outcomes, leaving this aspect still underexplored.

In light of our findings, we may hypothesize that surgery may still play a pivotal role in managing CF-CRS patients even in the ETI era, as it was proven to be safe and effective in improving quality of life, sense of smell, and radiological metrics in CF-CRS patients, regardless of genotype, nasal polyps, and previous ESS. Given the generally long interval observed between the last surgery and the beginning of triple therapy, we may cautiously speculate that ESS can offer durable sinonasal health and that we may use surgery with no fear of diminishing the potential benefits of a possible future ETI therapy. While waiting for prospective and match-paired studies, our results suggest ESS as the first-line treatment modality in patients suffering from severe sinonasal symptomatology or post-surgical complications such as mucoceles, and in patients who are not eligible for ETI because of genetic mutations different from ΔF508 or lung transplantation history.

To the best of our knowledge, the strengths of our study include the largest cohort of adult CF-CRS patients treated with ESS or ETI, as well as the first comparison of sinonasal-related outcomes between medical and surgical treatment modalities. Furthermore, this is the first study reporting a semi-objective evaluation of the sense of smell through an SSIT in patients affected by CF-CRS and undergoing ESS. The monocentric nature of the study guarantees greater homogeneity of clinical, diagnostic, and therapeutic protocols, minimizing inter-center and inter-observer variability. However, the baseline clinical differences between surgically and medically treated patients represented the major limitation of our study. We acknowledge that conducting a similar study may be logistically challenging, as having two comparable groups may be arduous, due to the exclusion of patients who have undergone pulmonary transplantation. Another key limitation is the absence of a reliable mean interval between ESS and ETI initiation across our cohort. Due to incomplete or missing temporal data in several medical records, we were unable to compute this value accurately. This hinders our ability to assess whether the timing of ESS relative to ETI initiation may influence sinonasal or systemic outcomes. Furthermore, only one patient initiated ETI within five years after ESS, which severely limits our ability to explore potential short-term interactions between surgery and subsequent ETI therapy. Secondary limitations may be ascribable to the low number of patients, the short follow-up period, the utilization of one single questionnaire to evaluate the quality of life with no division between SNOT-22 subdomains, and the use of one single smell test to assess olfactory function. Finally, the retrospective nature of the study represents an inherent limitation, as it restricts control over confounding variables, relies on the accuracy and completeness of existing medical records, and may introduce selection or recall bias. These factors can affect the robustness of the findings and limit the ability to draw definitive causal inferences. Prospective, longitudinal, future studies with larger cohorts, extended follow-up periods, more accurate data about the interval between surgery and ETI initiation, and more detailed assessments of quality of life and olfactory function are needed to elucidate the true effects of ETI on CF-CRS control and identify patients who may derive greater benefit from surgery. These studies could also help identify subgroups of patients who might benefit more from surgery. Additionally, it would be valuable to investigate whether there is a difference in the improvement in symptoms in patients who started ETI soon after surgery versus those who started it after a longer delay, as this could help determine the optimal sequencing of these treatments.

## 5. Conclusions

CF-CRS heavily impacts patients’ quality of life. Both ETI and ESS were demonstrated to be safe and effective in reducing the sinonasal burden of disease, improving quality of life, and restoring the sense of smell. We demonstrated that ESS provides long-lasting sinonasal health, without compromising the efficacy of ETI in future medical management, suggesting that the two treatment modalities can coexist. Importantly, our current data do not suggest that prior surgery either compromises or enhances the efficacy of ETI. Thus, the role of surgery in directly modifying ETI response still remains unclear. Even in the ETI epoch, surgery may still play a crucial role in managing CF-CRS patients, particularly in those not eligible for CFTR modulator therapy or experiencing severe disease not adequately controlled with medical therapy alone.

## Figures and Tables

**Table 1:** Demographic characteristics of enrolled patients according to their treatment. DM2: diabetes mellitus type 2; ESS: endoscopic sinus surgery; ETI: elexacaftor, tezacaftor, and ivacaftor.

	ESS n(%)	ETI n(%)			Overall n (%)
		Surgically Naive	Post-ESS	Overall	
**Sex**					
Male	17 (68.0)	7 (41.2)	21 (56.8)	28 (51.9)	45 (56.8)
Female	8 (32.0)	10 (58.8)	16 (43.2)	26 (48.1)	34 (43.2)
**Age**	39.5	37.4	34.4	35.1	36.3
**ΔF508 mutation**					
heterozygous	20 (80.0)	15 (88.2)	30 (81.1)	40 (81.6)	60 (75.9)
homozygous	5 (20.0)	2 (11.8)	7 (18.9)	9 (18.4)	14 (24.1)
**Lung Transplant**					
no	15 (60.0)	17	37	54	69 (87.3)
yes	10 (40.0)	0	0	0	10 (12.7)
**DM2**					
no	14 (56.0)	11 (64.7)	26 (70.3)	37 (68.5)	51 (64.6)
yes	11 (44.0)	6 (35.3)	11 (29.7)	17 (31.5)	28 (35.4)
**Airborne allergies**					
no	21 (84.0)	13 (76.5)	24 (64.9)	37 (68.5)	58 (73.4)
yes	4 (16.0)	4 (23.5)	13 (35.1)	17 (31.5)	21 (26.6)
**Previous ESS**					
no	16 (64.0)	17	0	17 (31.5)	33 (41.7)
yes	9 (36.0)	0	37	37 (68.5)	46 (58.3)

**Table 2:** Mean variations after one year in sinonasal scores. An * indicates a statistically significant change. SNOT-22: sinonasal outcome test-22; NPS: nasal polyps score; mLKS: modified Lund–Kennedy score; ESS: endoscopic sinus surgery; ETI: elexacaftor, tezacaftor, and ivacaftor.

	Pre-Treatment	Post-Treatment	Variation (*p*-Value)
**SNOT-22**			
ESS	40.1	12.1	−28.0 (*p* < 0.05) *
ETI	51.4	21.6	−29.8 (*p* < 0.05) *
Surgically naive ETI	44.3	24.3	−20.0 (*p* < 0.05) *
Post-ESS ETI	56.0	21.4	−34.6 (*p* < 0.05) *
Overall	48.7	19.0	−29.7 (*p* < 0.05) *
**NPS**			
ESS	4.3	1.3	−3.0 (*p* < 0.05) *
ETI	1.5	0.8	−0.7 (*p* < 0.05) *
Surgically naive ETI	0.9	0.4	−0.5 (*p* = 0.09)
Post-ESS ETI	1.7	0.9	−0.8 (*p* < 0.05) *
Overall	2.4	1.0	−1.4 (*p* < 0.05) *
**mLKS**			
ESS	6.3	3.2	−3.1 (*p* < 0.05) *
ETI	3.7	3.5	−0.2 (*p* = 0.698)
Surgically naive ETI	4.1	3.7	−0.4 (*p* = 0.683)
Post-ESS ETI	3.5	3.3	−0.2 (*p* = 0.795)
Overall	4.5	3.4	−1.1 (*p* = 0.01) *
**Sniff Test**			
ESS	6.4	9.8	+3.4 (*p* = 0.04) *
ETI	9.5	11.5	+2.0 (*p* < 0.01) *
Surgically naive ETI	9.7	12.1	+2.4 (*p* = 0.18)
Post-ESS ETI	9.3	11.3	+2.0 (*p* = 0.03) *
Overall	8.8	11.0	+2.2 (*p* = 0.01) *
**Lund–Mackay**			
ESS	15.6	6.7	−8.9 (*p* < 0.05) *
ETI	14.6	6.4	−9.2 (*p* < 0.05) *
Surgically naive ETI	10.1	4.0	−6.1(*p* = 0.14)
Post-ESS ETI	15.8	6.8	−9.0 (*p* < 0.05) *
Overall	14.8	6.3	−8.5 (*p* < 0.05) *

**Table 3:** Mean changes in sinonasal scores after 12 months in surgically treated and medically treated patients and between surgically naive and post-ESS ETI patients. An * indicates a statistically significant difference. SNOT-22: sinonasal outcome test-22; NPS: nasal polyps score; mLKS: modified Lund–Kennedy score; LMS: Lund–Mackay score; ESS: endoscopic sinus surgery; ETI: elexacaftor, tezacaftor, and ivacaftor.

	ESS	ETI	*p*-Value	Surgically Naive ETI	Post-ESS ETI	*p*-Value
**SNOT-22**	−28.0	−29.8	0.121	−20.0	−34.6	0.120
**NPS**	−3.0	−0.7	<0.05 *	−0.5	−0.8	0.532
**mLKS**	−3.1	−0.2	<0.05 *	−0.4	−0.2	0.490
**Sniff test**	+3.4	+2.0	0.229	+2.4	+2.0	0.835
**LMS**	−8.9	−9.2	0.957	−6.1	−9.0	0.076

## Data Availability

The data presented in this study are available on request from the corresponding author due to privacy.
